# The isolation and characterisation of the wheat molecular ZIPper I homologue, *Ta*ZYP1

**DOI:** 10.1186/1756-0500-5-106

**Published:** 2012-02-18

**Authors:** Kelvin HP Khoo, Amanda J Able, Jason A Able

**Affiliations:** 1 School of Agriculture, Food & Wine, Waite Research Institute, The University of Adelaide, Waite Campus, PMB1, Glen Osmond, South Australia 5064

## Abstract

**Background:**

The synaptonemal complex (SC) is a proteinaceous tripartite structure used to hold homologous chromosomes together during the early stages of meiosis. The yeast ZIP1 and its homologues in other species have previously been characterised as the transverse filament protein of the synaptonemal complex. Proper installation of ZYP1 along chromosomes has been shown to be dependent on the axial element-associated protein, ASY1 in Arabidopsis.

**Results:**

Here we report the isolation of the wheat (*Triticum aestivum*) *ZYP1 *(*TaZYP1*) and its expression profile (during and post-meiosis) in wild-type, the *ph1b *deletion mutant as well as in *Taasy1 *RNAi knock-down mutants. *Ta*ZYP1 has a putative DNA-binding S/TPXX motif in its C-terminal region and we provide evidence that *Ta*ZYP1 interacts non-preferentially with both single- and double-stranded DNA *in vitro*. 3-dimensional dual immunofluorescence localisation assays conducted with an antibody raised against *Ta*ZYP1 show that *Ta*ZYP1 interacts with chromatin during meiosis but does not co-localise to regions of chromatin where *Ta*ASY1 is present. The *Ta*ZYP1 signal lengthens into regions of chromatin where *Ta*ASY1 has been removed in wild-type but this appears delayed in the *ph1b *mutant. The localisation profile of *Ta*ZYP1 in four *Taasy1 *knock-down mutants is similar to wild-type but *Ta*ZYP1 signal intensity appears weaker and more diffused.

**Conclusions:**

In contrast to previous studies performed on plant species where ZYP1 signal is sandwiched by ASY1 signal located on both axial elements of the SC, data from the 3-dimensional dual immunofluorescence localisation assays conducted in this study show that *Ta*ZYP1 signal only lengthens into regions of chromatin after *Ta*ASY1 signal is being unloaded. However, the observation that *Ta*ZYP1 loading appears delayed in both the *ph1b *and *Taasy1 *mutants suggests that *Ta*ASY1 may still be essential for *Ta*ZYP1 to play a role in SC formation during meiosis. These data further suggest that the temporal installation of ZYP1 onto pairing homologous chromosomes in wheat is different to that of other plant species and highlights the need to study this synaptonemal complex protein on a species to species basis.

## Background

The generation of gametes in sexually-reproducing organisms occurs through the reductional division process of meiosis that involves a single DNA replication event followed by two consecutive cell division events resulting in the formation of four haploid gametes from a single diploid progenitor cell. During the early substages of prophase I, the homologous chromosomes roughly align and pair with one another while axial element (AE) components, such as the ASYnapsis 1 (ASY1) protein, are installed along the lengths of the paired homologues. During leptotene to zygotene, transverse filament (TF) proteins of the proteinaceous ultrastructure known as the synaptonemal complex (SC) are installed to span the gap between the AE backbones. The central region of the SC consists of the polymerising ends of the TF that interact with one another to hold the homologous chromosomes together. During pachytene, the chromosomes are completely synapsed with the SC completely installed throughout the lengths of the chromosomes. Disassembly of the SC during diplotene leaves the homologous chromosomes attached only by chiasmata formed through the genetic recombination cross-over process [1,2].

While the structure of the SC has been studied using cytology techniques extensively since the 1950s, the first plant synaptonemal complex (SC) proteins, ZYP1a and ZYP1b (collectively known as ZYP1) were only recently identified and characterised in *Arabidopsis thaliana*. These were named after the *Saccharomyces cereviseae *homologue, *Molecular ZIPper 1 *(*ScZIP1*). Characterisation of *Sc*ZIP1 previously revealed that its globular C-terminal domain interacts with the AE (also known as the lateral elements) while the N-terminus of the protein is able to dimerise with the N-termini of other ZIP1 molecules to form the central element of the SC [3]. In Arabidopsis, *AtZYP1a *and *AtZYP1b *arose from a gene duplication event and encode proteins that share structural and functional similarities to *Sc*ZIP1 [3,4]. Both *At*ZYP1a and *At*ZYP1b form the TF of the proteinaceous tripartite SC during early meiosis and are functionally redundant [4]. More recently, two other plant homologues of ZYP1 have also been studied in *Secale cereale *(*Sc*ZYP1) and *Oryza sativa *(*Os*ZEP1) [5,6]. Although the SC appears to be a well-conserved ultrastructure required for meiosis-specific chromosome pairing in various species, amino acid sequence conservation of its TF components across species is quite limited. A clear example of this can be seen in the ZIP1, SYP1, C(3)G, SCP1 and ZEP1 TF proteins of the SC that have previously been characterised in various species such as budding yeast, *Drosophilia melanogaster*, *Caenorhabditis elegans*, *Mus musculus*, and more recently, in some plant species such as *Arabidopsis thaliana *and *Oryza sativa *[1,2,4,6-12]. Although these proteins have limited conservation at both the DNA and amino acid sequence levels, they all share the same central α-helical coiled-coil tertiary structure capped at both termini with globular domains and perform an identical function within their respective species [10].

In plants, the immuno-localisation profiles of the ZYP1 TF homologues also differ slightly from species to species, with ZYP1 signal first appearing as foci in leptotene stage meiocytes of Arabidopsis and rice but as short linear tracts in rye [4-6]. These ZYP1 foci are only observed after ASY1 has been loaded onto the AE backbones of the chromatin [4,5]. Using the Arabidopsis *spo11 *and *dmc1 *mutants, Higgins and colleagues [4] deduced that ZYP1 was recruited to double-strand break (DSB) sites during the early stages of single-stranded DNA invasion and that ZYP1 loading was independent of recombination initiation. However, lengthening of the ZYP1 signal along the chromatin was dependent on successful recombination which in turn relied on ASY-mediated loading of the DMC1 recombinase onto chromatin [13]. In rice and Arabidopsis, the ZYP1 signal lengthens from zygotene to pachytene and localises to the central region of the SC where it is sandwiched between ASY1 signals that are associated with the AE backbones of the homologous chromosomes on both sides [4,6].

Given that the synaptonemal complex is a well-conserved structure present in many species and the fact that ASY1 has been shown to be necessary for synapsis and homologous chromosome pairing in bread wheat [14,15], a ZYP1-like protein is also likely to be involved in these processes during the early stages of wheat meiosis. Indeed, knock-out of the ZYP1 protein in Arabidopsis leads to non-homologous interactions and the formation of bivalents/multivalents. Such abnormal chromosome interactions have also been observed in *Taasy1 *RNAi knock-down mutants as well as in the wheat pairing homologous (*Ph*) deletion mutant, *ph1b *[14].

The rice ZIP1 homologue (*Os*ZEP1) was also shown to perform similar roles to that of *At*ZYP1 [6]. However, unlike other ZYP1 homologues, ZEP1 was shown to be reloaded two more times onto decondensed chromatin after the diakinesis stage of prophase I, specifically during the prophase II and telophase II stages. Based on their observations, Wang and colleagues [6] inferred that ZEP1 may also maintain chromatin in a decondensed state during early microsporogenesis.

The functional differences and limited sequence conservation seen in the SC TF proteins of various species highlight the need to study these proteins on a species-specific basis to determine whether these proteins may have additional roles to their already well-characterised TF role. Here we report the isolation of the *TaZYP1 *coding sequence, analysis of its predicted amino acid sequence and the stage-specific *TaZYP1 *gene expression profiles in both wild-type and the *ph1b *mutant. We show that *Ta*ZYP1 interacts with both single- and double-stranded DNA *in vitro *and also report on the temporal and spatial localisation profile of *Ta*ZYP1 within wheat meiocytes of two wild-type cultivars and the *ph1b *mutant using 3-dimensional dual immuno-fluorescence localisation techniques. Due to the importance of ASY1 in synapsis, maintenance of homologous chromosome interactions, and its reported role in the complete installation of ZYP1 along the lengths of Arabidopsis chromosomes, the localisation profile of *Ta*ZYP1 was also investigated in four *Taasy1 *RNAi knock-down mutants.

## Results

### *TaZYP1 *encodes a predicted protein product with a putative S/TPXX DNA binding motif at the C-terminus

**Figure 1:**
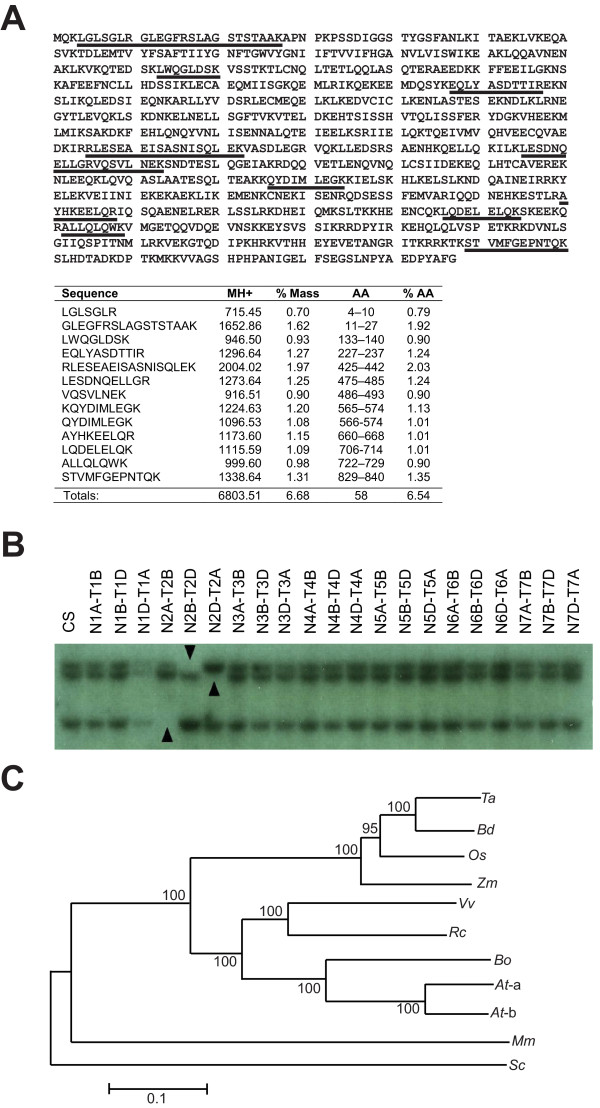
***Ta*ZYP1 shares sequence similarities withother plant species and is located on chromosome group 2 in wheat**. (A) Peptides identified from the MS/MS data of the digested heterologously-expressed *Ta*ZYP1 protein that were identical to those within the rice *Os*ZYP1 sequence [TIGR rice locus identifier: LOC_Os04g37960] are highlighted in the amino acid sequence (red font). (B) Membranes prepared with DNA from nullisomic (N)-tetrasomic (T) wheat lines of Chinese Spring (CS) were hybridised with a *TaZYP1*-specific probe showing that *TaZYP1 *is located on chromosome group 2 with a copy on the A, B and D genomes (indicated by black arrowheads). The faint signal in lane 4 (N1D-T1A) was caused by uneven loading. (C) The evolutionary history of *Ta*ZYP1 and its homologues was inferred using the Neighbor-Joining method. The bootstrap consensus tree inferred from 10000 replicates is taken to represent the evolutionary history of the 11 taxa analysed. Branches corresponding to partitions reproduced in less than 50% bootstrap replicates are collapsed. The percentages of replicate trees in which the associated taxa clustered together in the bootstrap test (10000 replicates) are shown next to the branches. The tree is drawn to scale, with branch lengths in the same units as those of the evolutionary distances used to infer the phylogenetic tree. All positions containing alignment gaps and missing data were eliminated only in pairwise sequence comparisons. There were a total of 1019 positions in the final dataset. Phylogenetic analysis was conducted using MEGA4. Ta-*Triticum aestivum*; Bd-*Brachypodium distachyon*; Os-*Oryza sativa*; Zm-*Zea mays*; Vv-*Vitus vinifera*; Rc-*Ricinus communis*; Bo-*Brassica oleracea*; At-a-*Arabidopsis thaliana *ZIP1a; At-b-*Arabidopsis thaliana *ZIP1b; Mm-*Mus musculus*; Sc-*Saccharomyces cereviseae*.

The full-length 2592 bp coding sequence of *TaZYP1 *was isolated using a combination of standard and 3' RACE PCR techniques. This transcript encodes a protein product 863 aa in length (Figure 1A) with a predicted MW of 98.541 kDa and pI of 6.4. Southern blot analysis using membranes prepared with digested DNA from nullisomic-tetrasomic wheat plants showed that the *TaZYP1 *gene is located on chromosome group 2 with a copy on the A, B and D genome respectively (Figure 1B).

The predicted *Ta*ZYP1 amino acid sequence shares high levels of conservation with its homologues in close relatives, including the rice transverse element protein [GenBank: ADD69817] (Identities = 80%, Positivies = 91%, *E*-value = 0.00) and maize ZYP1 [GenBank: HQ116413] (Identities = 75.9%, Positives = 88%, *E*-value = 0.0) (Figure 1C). However, the level of sequence conservation was reduced when compared with the Arabidopsis homologues, *At*ZYP1b [GenBank: NP_564164.1] (Identities = 40%, Positives = 64%, *E*-value = 1e-156) and *At*ZYP1a [GenBank: NP_173645.3] (Identities = 39%; Positives = 63%, *E*-value = 1e-152) and even further reduced in species from other kingdoms as shown in the phylogenetic analysis (Figure 1C).

Amino acid sequence analysis revealed that both the N- and C-terminal regions of *Ta*ZYP1 (aa positions 1-68 and 723-863 respectively) have high pI values of 10.22 and 10.05. In addition, 18.75% of the C-terminus consisted of arginine and lysine residues. A putative DNA-binding S/TPXX motif was also found within this region (aa positions 761 to 764) (Figure 1A). Conserved domain analysis revealed that the *Ta*ZYP1 amino acid sequence retained modest similarities with two known Structural Maintenance of Chromosomes (SMC) conserved domains characterised in archaea, namely SMC_prok_B.and SMC_prok_A (*E*-values = 3.65e-09 and 3.00e-04 respectively) (data not shown). 3-dimensional protein modelling predicted that the central region of *Ta*ZYP1 forms a coiled-coil domain structure (from aa position 69 to 722) (data not shown).

### *Ta*ZYP1 interacts with DNA and is up-regulated during early meiosis

**Figure 2:**
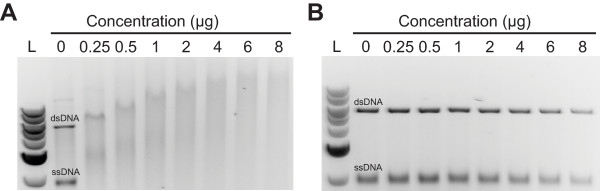
***Ta*ZYP1 interacts with both single- and double-stranded DNA *in vitro***. *Ta*ZYP1 interactions with both DNA species appear equal as the severity of retardation of both DNA species appears identical in each lane regardless of the concentration of *Ta*ZYP1 present. (A) Full-length *Ta*ZYP1 protein extracted under native conditions; (B) Total cell protein extract from non-induced control cell line.

Because other ZYP1 proteins have been shown to interact with DNA during the formation of the SC and the presence of the S/TPXX DNA-binding motif found in the C-terminal region of *Ta*ZYP1, the full-length *Ta*ZYP1 protein was used in competitive DNA-binding assays to investigate whether *Ta*ZYP1 interacted with DNA. The results showed that the *Ta*ZYP1 protein interacted with both single-stranded DNA (ssDNA) and double-stranded DNA (dsDNA) without any preferences as evidenced by the equal rates of retardation of both DNA species regardless of the *Ta*ZYP1 concentration used in the assays (Figure 2).

**Figure 3:**
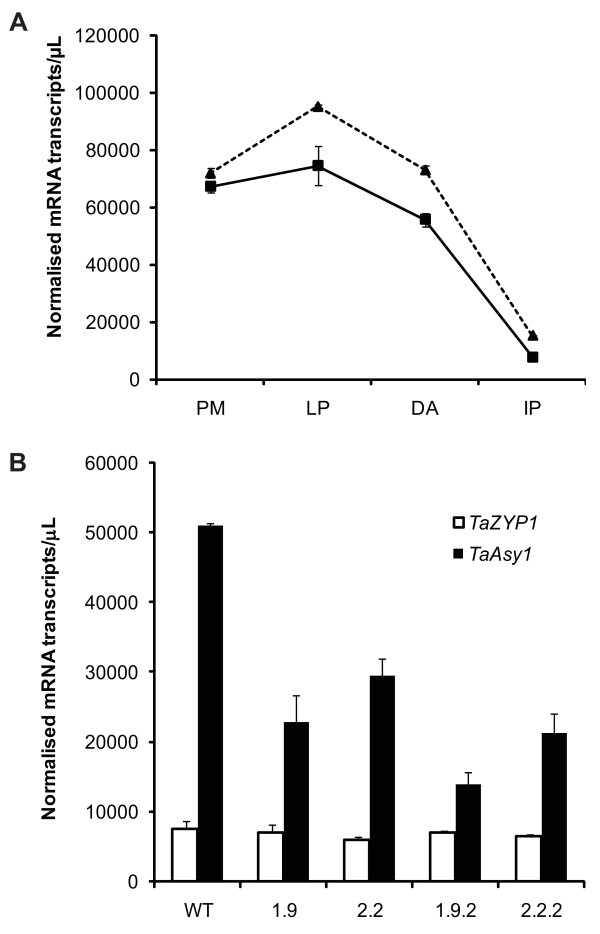
***TaZYP1 *is upregulated during early meiosis and is affected in the *ph1b *mutant**. (A) Q-PCR analysis of *TaZYP1 *transcript numbers revealed that *TaZYP1 *is highly expressed during the early stages of meiosis but reduces significantly at the later stages examined in Chinese Spring wild-type (solid line). While the general expression profile of *TaZYP1 *in the *ph1b *mutant (dotted line) is similar to that of the wild-type, *TaZYP1 *transcript numbers are elevated with a 1.3-fold increase seen during leptotene to pachytene when compared to the wild-type. PM-pre-meiotic interphase; L-P-leptotene to pachytene pooled stage; D-A-diplotene to anaphase I pooled stage; IP-immature pollen. (B) No statistically-significant differences in expression levels of *TaZYP1 *were observed between Bob White MPB26 (BW) wild-type and the *Taasy1 *mutants. Error bars represent the mean ± standard error of three replicate experiments.

Quantitative Real-Time PCR (Q-PCR) profiling was conducted to determine the transcript expression levels of *TaZYP1 *across various stages of meiosis in both the Chinese Spring wild-type and the *ph1b *mutant (Figure 3A). *TaZYP1 *was highly expressed during pre-meiotic interphase. This high level of *TaZYP1 *expression persisted as the cells progressed through the early stages of meiosis up to pachytene but transcript numbers then steadily declined from diplotene to anaphase I. By the stage of immature pollen formation, *TaZYP1 *transcript levels were only approximately 11% that of the pre-meiotic interphase stage transcript levels. Based on work conducted by Boden and colleagues [14] showing that *TaASY1 *is up-regulated in the *ph1b *mutant and observations in Arabidopsis that ASY1 is required for correct localisation of ZYP1 during early meiosis [13], *TaZYP1 *transcript levels were also investigated in the *ph1b *mutant and four *Taasy1 *knock-down mutants. In the *ph1b *mutant, *TaZYP1 *transcripts were present at increased levels throughout all stages of meiosis examined but the overall expression pattern of *TaZYP1 *remained similar to that of wild-type. The largest differences in *TaZYP1 *transcript copy numbers were seen in the leptotene-pachytene (L-P) and the diplotene-anaphase I (D-A) pooled stages with an approximate 1.27-fold and 1.30-fold increase seen in the *ph1b *mutant. In contrast, Q-PCR analysis of the four individual RNAi knock-down *Taasy1 *mutant lines investigated (two from the T_1 _generation and two from the T_2 _generation) revealed no statistically-significant difference (p < 0.05) between the expression levels of *TaZYP1 *in the mutants compared to wild-type even though the *TaASY1 *transcript levels were significantly lower in all four *Taasy1 *mutants (Figure 3B).

### *Ta*ZYP1 is loaded onto regions of chromatin after *Ta*ASY1 is removed

**Figure 4:**
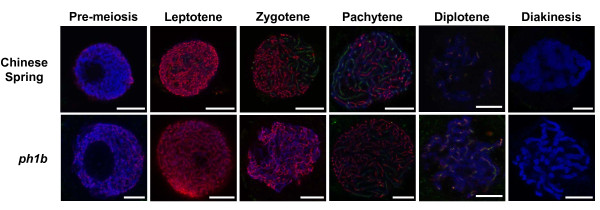
***Ta*ZYP1 localisation during sub-stages of prophase I in wild-type and *ph1b *mutant bread wheat**. In Chinese Spring wild-type meiocytes, *Ta*ZYP1 (green) first appears as faint punctuate foci that localise to chromatin during leptotene. *Ta*ZYP1 signal expands from these foci to form short tracts in zygotene before lengthening and condensing to form long thick tracts along the synapsing homologous chromosomes during pachytene. The *Ta*ZYP1 signal diminishes significantly by diplotene and is absent in diakinesis. Spatial localisation of *Ta*ZYP1 in the *ph1b *mutant was similar to wild-type but differed temporally. The *Ta*ZYP1 signal remained as punctuate foci until the zygotene-pachytene transition before lengthening of the signal into short tracts were observed. The signal of *Ta*ZYP1 also persisted into diplotene at higher levels when compared to wild-type. *Ta*ASY1 (red) was used as a marker of early meiotic events. Chromatin (blue) was counter-stained with DAPI. Scale bars, 7.5 μm.

3D-immunolocalisation of *Ta*ZYP1 in wild-type wheat meiocytes showed that *Ta*ZYP1 is localised to regions of chromatin during early meiosis (Figure 4; row 1, columns 1-6). *Ta*ZYP1 was first observed as faint punctate foci that localised to chromatin during early-leptotene (Figure 4; row 1, panel 2). These foci then formed short linear tracts along the chromatin as the cells progressed into zygotene (Figure 4; row 1, panel 3). From mid-zygotene to pachytene, the *Ta*ZYP1 signal continued to lengthen but also condensed mirroring the condensation of the chromatin. During late pachytene, the *Ta*ZYP1 signal appeared as long tracts that populated regions of chromatin even after *Ta*ASY1 was unloaded (Figure 4; row 1, panel 4). The *Ta*ZYP1 signal then quickly dissipated from late-pachytene to diplotene where only low residual levels of *Ta*ZYP1 signal were observed (Figure 4; row 1, panel 5). No *Ta*ZYP1 signal was observed during diakinesis (Figure 4; row 1, panel 6). Interestingly, the *Ta*ZYP1 signal appeared on regions of chromatin only after *Ta*ASY1 first appeared within those regions and only lengthened into those regions after *Ta*ASY1 signal was unloaded from them (Figure 4; row 1, panels 1-6). Although *Ta*ZYP1 and *Ta*ASY1 signals were sometimes seen localised to the same regions of chromatin simultaneously, they did not co-localise with one another.

Having determined that *TaZYP1 *expression levels were slightly elevated in the *ph1b *mutant (Figure 3A), dual immunofluorescence localisation assays were performed to determine whether *Ta*ZYP1 loading was affected within the *ph1b *mutant. As previously reported by Boden *et al*. [14], the *Ta*ASY1 signal was observed to be stronger in the *ph1b *mutant with the nucleoplasm of some observed meiocytes completely saturated even at pre-meiotic interphase (data not shown). Furthermore, the *Ta*ASY1 signal appeared disordered on the condensing chromatin and persisted into diplotene. Aggregates of *Ta*ASY1 protein, thought to be polycomplexes, were sometimes observed as large foci in nucleolar regions of the *ph1b *meiocytes. Similar to wild-type, *Ta*ZYP1 was first observed as faint punctate foci during leptotene in the *ph1b *mutant (Figure 4; row 2, panel 2). However, in contrast to wild-type, lengthening of the signal from foci to form short tracts was delayed until pachytene (Figure 4; row 2, panel 4). In addition, *Ta*ZYP1 signal persisted at higher residual levels in diplotene compared to wild-type, possibly as a consequence of the delayed loading of *Ta*ZYP1 in the *ph1b *mutant (Figure 4; row 2, panel 5).

### *Ta*ZYP1 signal along chromosomes in the *Taasy1 *knock-down mutants appear similar to wild-type albeit weaker and more diffuse

**Figure 5:**
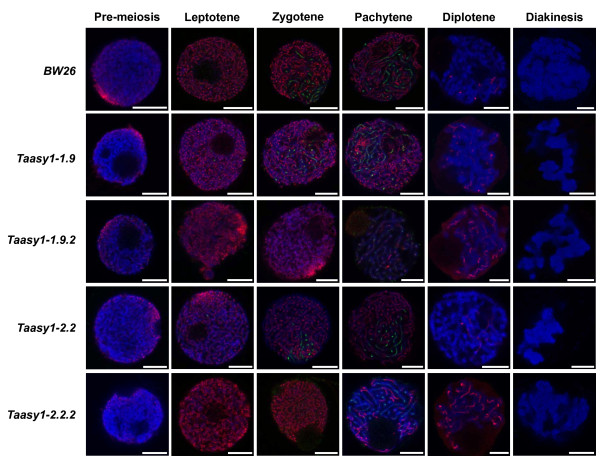
**Localisation of *Ta*ZYP1 and *Ta*ASY1 across four *Taasy1 *RNAi knock-down mutants**. In general, *Ta*ZYP1 (green) localisation within the *Taasy1 *mutants was similar to that of wild-type with lengthening of the *Ta*ZYP1 signal observed as diffuse disrupted tracts during pachytene. Compared to wild-type, *TaASY1 *(red) signals were diffuse and slightly weaker in the mutants and persisted at higher levels through to diplotene. Chromatin (blue) was counter-stained with DAPI. Scale bar, 7.5 μm.

Dual immunofluorescence localisation assays were also performed on meiocytes of four *Taasy1 *knock-down mutant lines and the isogenic wild-type Bob White MPB26 (Figure 5). In general, *Ta*ASY1 signals in the mutant lines appeared similar to wild-type both spatially and temporally but appeared to be weaker, diffuse and disrupted. In addition, *Ta*ASY1 persisted at much higher levels during diplotene in the *Taasy1 *mutants (Figure 5; rows 2-5, panel 5) compared to the wild-type (compare Figure 5; row 1, panel 5).

*Ta*ZYP1 localisation was identical both temporally and spatially in the Bob White MPB26 and Chinese Spring wild-types (compare Figure 5; row 1 with Figure 4 row 1). Similar to observations of both wild-type meiocytes (Figures 4 and 5; row 1), *Ta*ZYP1 and *Ta*ASY1 signals were sometimes seen populating regions of chromatin simultaneously but were never co-localised. Amongst the *Taasy1 *mutants, *TaZYP1 *signals were generally similar to wild-type, first appearing as faint punctate foci on regions of chromatin during leptotene and lengthening into short tracts during zygotene (Figure 5; rows 2 to 5, panels 1 to 3). The *Ta*ZYP1 signal then condensed and lengthened further during pachytene, populating regions of chromatin where the *Ta*ASY1 signal was being unloaded (Figure 5; rows 2 to 5, panel 4). Of the *Taasy1 *mutants analysed, ZYP1 loading in the *Taasy1-1.9.2 *mutant during pachytene appeared to be weaker, more diffuse and disrupted in addition to showing a delayed lengthening of the ZYP1 signal during the zygotene stage compared to wild-type (Figure 5; row 3, panel 3).*Ta*ZYP1 signal in the *Taasy1 *mutants was only present at residual levels during diplotene comparable to levels seen in the wild-type (compare Figure 5; row 1, panel 5 with *Taasy1 *mutant panels down column 5).

## Discussion

This study has characterised the ZYP1 bread wheat homologue at both the genetic and protein levels. Characterisation of the *Ta*ZYP1 amino acid sequence revealed that it shares similar physiochemical properties with its previous characterised homologues in other species with predictive modeling of *Ta*ZYP1 showing that it is a coiled-coil protein, capped with globular domains on both ends and a C-terminal region that possesses a high pI and a S/TPXX DNA-binding motif. Expression profiling of *TaZYP1 *revealed that *TaZYP1 *transcript numbers are elevated from pre-meiotic interphase to pachytene before reducing from diplotene onwards. Competitive DNA-binding assays confirm that *Ta*ZYP1 interacts with both ss- and ds-DNA indiscriminately *in vitro *with data from 3-dimensional fluorescence localisation assays conducted with an anti-*Ta*ZYP1 polyclonal antibody further confirming that *Ta*ZYP1 interacts with chromatin during the early stages of meiosis. *Ta*ZYP1 was localised to chromatin from leptotene to pachytene and while the *Ta*ZYP1 foci signal was sometimes observed to be present on the same regions of chromatin as the *Ta*ASY1 signal, both signals did not co-localise. In general, *Ta*ZYP1 signal only lengthened into regions of chromatin after the *Ta*ASY1 signal was unloaded. In the *ph1b *mutant, lengthening of the *Ta*ZYP1 signal appeared delayed until pachytene and persisted at higher levels in diplotene compared to wild-type. Localisation of *Ta*ZYP1 in four *Taasy1 *mutants revealed that *Ta*ZYP1 loading was similar to wild-type but most severely affected in the *Taasy1-1.9.2 *mutant (which had the lowest *TaASY1 *expression).

In order to characterise the wheat ZYP1 homologue, the full-length coding sequence of the wheat *ZYP1 *homologue was isolated using sequence information from a rice EST that showed high levels of similarity to the functionally-redundant *AtZYP1a *and *AtZYP1b *genes [4]. However, unlike Arabidopsis [4], allohexaploid bread wheat appears to have only one *ZYP1 *gene, with copies on chromosome 2A, B and D. This was not unexpected as Higgins *et al*. [4] previously hypothesised that the two Arabidopsis *ZYP1 *genes were caused by a duplication event that occurred after the divergence of the Arabidopsis and brassica genera and that the *AtZYP1 *genes were more closely-related to each other than they were to the only *ZYP1 *homologue found in *Brassica oleracea*. More recent data supports this hypothesis, as both *Oryza sativa *and *Zea mays *have been reported to possess only a single *ZYP1 *homologue [6,16]. Although *TaZYP1 *has been physically mapped to chromosome group 2 using the nullisomic/tetrasomic Southern blot assays, further research must be conducted to determine which orthologue of *TaZYP1 *has been isolated and whether its orthologues on the other genomes are transcriptionally active as well as whether their protein products are functionally redundant. The observation that the sequence of ZYP1 was not highly-conserved between other non-plant species was not unexpected as SC proteins have been previously reported to share conserved function but lack sequence conservation [4].

ZYP1 proteins form rod-like homodimers that are capped at the ends by globular N- and C-termini with the globular N-terminus necessary for ZYP1 homodimer formation [3]. ZYP1 molecules attach to the lateral elements of sister chromosomes via their C-termini and extend outward allowing the N-termini to interact with one another thus forming the central element of the SC [reviewed by 10; and references therein]. Amino acid sequence analysis of *Ta*ZYP1 revealed that both the N and C-termini of the protein are basic with high pI values, while predictive protein modeling revealed that the central region of *Ta*ZYP1 has a coiled-coil central region. The high pI value of the C-terminus is particularly interesting as it may allow the lysine and arginine residues within it to bind DNA when the physiological pH of the cell is more acidic than the pI of the C-termini (which is 10.03). Positively-charged lysine and arginine residues have previously been shown to bind DNA in a non-specific manner in leucine zipper and helix-loop-helix motifs [17,18]. In addition, the presence of a S/TPXX motif, previously characterised by Suzuki [19], within the *Ta*ZYP1 C-terminus provides further evidence that the *Ta*ZYP1 should also have a similar localisation pattern and arrangement within the SC as its previously characterised homologues. Furthermore, cDART database searches with *Ta*ZYP1 amino acid sequence returned moderate hits to two partial SMC conserved domains previously characterised in archaea (data not shown). Both these domains have previously been reported in archaea proteins that interact with DNA [20,21], and these domains also appear to be present in higher eukaryotic species [22].

To determine whether *Ta*ZYP1 binds to DNA, purified *Ta*ZYP1 protein heterologously-expressed in *E. coli *was used in competitive DNA-binding assays. The equal levels of retardation of both the ss- and dsDNA species indicates that *Ta*ZYP1 is able to bind both species of DNA indiscriminately under *in vitro *conditions (Figure 2). The observation that *Ta*ZYP1 is able to interact with both ss- and dsDNA was not unexpected as studies in both mice and *C. elegans zyp1 *homologue deletion mutants have shown that the recruitment of the ZYP1 homologues to DNA recombination sites is essential for the unloading of the recombination machinery during meiosis within both organisms [7,23]. Under wild-type conditions in mice and *C. elegans*, loading of the ZYP1 signal precedes the removal of the RAD51 foci signal (thus indicating that ZYP1 recruitment to regions of chromatin with RAD51 recombinase, and hence regions of DNA where single-stranded DNA invasion have occurred, is required before the removal of the recombination machinery). Furthermore, work conducted by Higgins and colleagues [4] on the Arabidopsis *spo11 *and *dmc1 *knock-out mutants show that the Arabidopsis ZYP1 homologues failed to localise to chromatin in the *spo11 *mutant but were localised as foci to sites of unsynapsed chromosome axes in the *dmc1 *mutant. Taken together, these data indicate that ZYP1 localisation to chromatin in Arabidopsis is dependent on double-stranded break (DSB) formation but independent of recombination initiation, thus inferring that ZYP1 is most likely recruited to sites of recombination during the very early stages of single-strand DNA invasion while ZYP1 polymerisation along the length of the chromosomes is dependent on recombination occurring [4].

Q-PCR profiling showed that *TaZYP1 *expression exhibits the profile of a protein typically required during early meiosis (Figure 3A). The relatively high expression level during the stages of pre-meiosis through to pachytene correlated well with observations in the immunofluorescence localisation assays where *Ta*ZYP1 signal was first observed in leptotene and peaked at pachytene (Figures 4 and 5; row 1). The reduction in expression post-pachytene was also reflected in the immunolocalisation loading with the *Ta*ZYP1 signal quickly diminishing post-pachytene (Figures 4 and 5; row 1). Comparisons of the *TaZYP1 *expression profiles between the wild-type and *ph1b *mutant showed that while the overall expression profiles were similar, transcript levels were elevated in the *ph1b *mutant at all stages examined (Figure 3A). Whether this increased *TaZYP1 *expression can be linked to the deletion of the *Ph1 *locus or whether it is a physiological reaction to the increased *Ta*ASY1 protein levels within the *ph1b *mutant, is yet to be determined. However, *TaZYP1 *expression was not affected in the *Taasy1 *mutants (Figure 3B).

Although *Ta*ZYP1 and *Ta*ASY1 were both present on chromatin, they did not co-localise (Figures 4 and 5). Even in the few instances where both signals were observed on the same regions of chromatin simultaneously, no co-localisation signals were detected. This observation indicates that *Ta*ZYP1, like its homologues in other species [4-6], appears to be a transverse filament protein and would therefore not be expected to co-localise with *Ta*ASY1 (which associates with the axial and lateral elements of chromatin). However, we also observed *Ta*ZYP1 signal being loaded onto regions of chromatin where *Ta*ASY1 was being unloaded. Interestingly, this aspect of the *Ta*ZYP1 localisation pattern is in contrast to the localisation patterns of its homologues in *Arabidopsis thaliana *and *Secale cereale*, where the ZYP1 homologues are load onto the chromatin while ASY1 signal is still present, forming a co-aligned ZYP1 signal that is sandwiched between the ASY1 signal associated to axial elements on both sides [4,5]. The delayed lengthening of the *Ta*ZYP1 signal along the chromatin may suggest that *Ta*ZYP1 could have an additional and as yet undefined role during the process of chromosome synapsis or that chromosome synapsis in bread wheat occurs through a slightly different mechanism compared to other plant species. It is also noteworthy that the rye ZYP1 homologue signal already appears as linear tracts during leptotene [5], unlike the punctate foci observed during the leptotene stage of both Arabidopsis [4], rice [6] and bread wheat in this study. This clearly shows that differences are present within the temporal loading patterns of the ZYP1 protein in different species.

The presence of relatively high levels of *Ta*ASY1 signal in meiocytes of the *ph1b *and *Taasy1 *mutants during diplotene (Figure 4; row 2 and Figure 5; rows 2-5) is also of interest as the Arabidopsis ZYP1 signal fails to lengthen in the *asy1 *knock-down mutant, possibly indicating that ASY1 may directly/indirectly mediate ZYP1 loading onto chromatin [13]. These increased levels of *Ta*ASY1 observed are probably the result of different mechanisms; as *Ta*ASY1 protein levels are significantly higher in the *ph1b *mutant but significantly lower in the *Taasy1 *knock-down mutants [14]. Boden and colleagues [14] previously hypothesised that the *Ta*ASY1 signal seen in *ph1b *diplotene meiocytes could be a result of too much *Ta*ASY1 protein present within the cells thus requiring more time for the cellular machinery to unload the *Ta*ASY1 molecules which leads to persistent *Ta*ASY1 signal in later stages of meiosis. In contrast, the persisting *Ta*ASY1 signal in the *Taasy1 *mutants could be explained by a cellular compensation mechanism that is delaying the unloading of the *Ta*ASY1 protein. Previous work in *Taasy1 *and *asy1 *homologue mutants have shown that chromosomes in these mutants lack a true pachytene stage and that levels of chromosome synapsis were reduced [14,24,25]. Perhaps cell cycle check-point mechanisms are trying to lengthen the duration for which *Ta*ASY1 is loaded onto the axial elements to allow proper synapsis to occur along the lengths of the homologous chromosomes. This then raises the question of whether *Ta*ZYP1 loading is completed within the *Taasy1 *mutants due to presence of *Ta*ASY1 signal in the diplotene stage where *Ta*ZYP1 signal was no longer observed. Work conducted in the Arabidopsis *asy1 *mutant previously showed that the *At*ZYP1 homologues are loaded onto the chromatin as foci during leptotene but subsequently fail to lengthen into linear tracts [13]. This again is at odds with the observations in this study which clearly show that the *Ta*ZYP1 signal still forms linear tracts in the *Taasy1 *mutants analysed. One possible explanation for this is the variation in severity of the *asy1 *knock-down effect in the mutants [14,26]. While the *ASY1 *gene expression data reported in both species are incomparable due to different gene expression analysis techniques being used, inferences could be made about the severity of the *ASY1 *knock-down effect by analysis of the fertility levels reported. While the Arabidopsis *asy1 *mutant has a more severe knock-down effect with only 10% fertility, the average fertility of the *Taasy1 *T_2 _mutants was 77.74%. This possibly indicates that higher levels of ASY1 protein were present in the *Taasy1 *mutants compared to that in the *Atasy1 *mutant thus leading to a less severe effect on *Ta*ZYP1 loading in the *Taasy1 *mutant meiocytes.

## Conclusions

In conclusion, this study has isolated and characterised the bread wheat ZYP1 homologue to further the current knowledge-base of bread wheat meiosis as well as to determine the role of ZYP1 in a complex hexaploid. Characterisation of *Ta*ZYP1 *in vitro *determined its DNA-binding capabilities while dual immunofluorescence localisation experiments with a polyclonal anti-*Ta*ZYP1 antibody on meiocytes of Chinese Spring and Bob White MPB26 wild-types as well as the *ph1b *and four *Taasy1 *mutants detailed its localisation profile during the early stages of meiosis both temporally and spatially. The localisation profile of *Ta*ZYP1 showed both similarities as well as differences with its previously characterised homologues in other species highlighting the need to further study this protein on a species-specific basis. While characterisation of *Ta*ZYP1 as a component of the SC has been achieved, further work is required to fully unravel the localisation profile of *Ta*ZYP1 and its other possible functions during the early stages of meiosis in bread wheat given the delayed lengthening of the *Ta*ZYP1 localisation signal with respect to *Ta*ASY1, in both wild-type and mutants (*ph1b *and *Taasy1*), compared to the localisation profiles of ZYP1 homologues previously reported in other species.

## Methods

### Plant material

Hexaploid wheat (*Triticum aestivum *L.) cv. Chinese Spring plants, four *Taasy1 *RNAi knock-down mutants (*Taasy1-1.9*, *Taasy1-1.9.2*, *Taasy1-2.2*, and *Taasy1-2.2.2*) and a Chinese Spring mutant lacking the *Ph1 *locus (*ph1b*) were grown in a glasshouse with a 16/8 h photoperiod at 23°C. Harvesting and staging of meiotic anthers from both wild-type and mutant plants, for quantitative real-time PCR (Q-PCR) and fluorescence immunolocalisation, were conducted as per Boden *et al*. (2009). Whole meiotic spike tissue was collected for the isolation and amplification of the cDNA *Triticum aestivum ZYP1 *(*TaZYP1*) sequence.

### RNA isolation and cDNA synthesis

Collected tissues for RNA isolation were initially ground in liquid nitrogen. Total RNA was extracted using Trizol reagent (Gibco-BRL, Carlsbad, CA, USA) according to the manufacturer's instructions. RNA concentration was determined using a Nanodrop (ND-1000) (Nanodrop, Wilmington, DE, USA). Total cDNA libraries for the wild-type and all the mutants analysed were synthesised from 2 μg of total RNA using the iScript cDNA synthesis kit (Bio-Rad, Hercules, CA, USA) according to the manufacturer's instructions. A Chinese Spring 3' RACE library was also synthesised from 2.5 μg of Chinese Spring total RNA per reaction using a GeneRacer Kit (Invitrogen) according to the manufacturer's instructions.

### cDNA amplification and sequencing of the ZYP1 coding sequence

Primers (see Additional file 1: Table S1) for isolating the *TaZYP1 *ORF were designed using the *OsZYP1 *sequence [TIGR Rice Annotation number: LOC_Os04g37960] (that showed sequence similarity to the Arabidopsis *ZYP1a *and *ZYPb *sequences) identified through a TIGR rice expressed sequence tag (EST) BLAST search (accessed 21^st ^October 2008).

Each PCR contained 100 ng cDNA, 0.2 mM dNTPs, 0.2 μM primers, 1U FastStart high fidelity *Taq *polymerase (Roche Applied Science, Mannheim, Germany) in 25 μL of 1× high fidelity buffer supplemented with 1× GC-RICH solution (Roche). PCR products were cloned into pCR8/GW/TOPO (Invitrogen) for DNA sequencing (15× coverage). Sequencing PCR was conducted using the GW1 and GW2 primers (see Additional file 1: Table S1). Secondary sets of primers were designed on the sequenced products to specifically amplify the *TaZYP1 *ORF. Amplification and sequencing was repeated as above. PCR cycling parameters were denaturation at 95°C for 5 min, followed by 35 cycles of 96°C for 30 s, T_m_°C for 30 s, 72°C for 2 min, with a final elongation step at 72°C for 10 min (see Additional file 1: Table S1 for T_m _of primer sets). The assigned NCBI accession number for *TaZYP1 *is JN129259.

### Bioinformatics analysis

DNA sequence alignments and comparisons were conducted with AlignX and Contig Express (Informax, VNTI Advance, Version 11, Frederick, MC, USA) software programs. VNTI software was also used to predict the molecular weight and pI of the protein. Detection for conserved domains was performed using the NCBI Conserved Domain Search Tool (http://www.ncbi.nlm.nih.gov/Structure/cdd/wrpsb.cgi), InterPro Scan (http://www.ebi.ac.uk/Tools/InterProScan/) and Pfam 23.0 (http://pfam.janelia.org/). Amino acid alignments and comparisons of full-length ZYP1 sequences (obtained from various BLAST searches using the NCBI, TIGR, and PredictProtein [http://www.predictprotein.org/;[27]] databases), and subsequent construction of the phylogenetic tree (neighbour-joining method) [28] was completed using Molecular Evolutionary Genetics Analysis (MEGA) software (version 4.0) [29]. Default parameters were used except for the following: the pair-wise deletion option was used, the internal branch test bootstrap value was set at 10,000 re-samplings, and the model setting was amino acid: Poisson correction with predicted gamma parameters set at 2.0. Accession numbers of the sequences used were: *Ta *- *Ta*ZYP1 [GenBank: JN129259]; *Bd *[Phytozome: Bradi5g12010]; *Os *[TIGR Rice Annotation Number: LOC_Os04g0452500]; *Zm *[GenBank: HQ116413]; *Rc *[GenBank: XP_002513917]; *Vv *[UniProt/TrEMBL: CBI19158.1]; *At-a *[GenBank: NP_173645]; *At-b *[GenBank: NP_564164]; *Bo *[GenBank: ABO69625]; *Mm *[GenBank: NP_035646]; and *Sc *[GenBank: NP_013498].

### Southern blot hybridisation

A 561 bp fragment of the *TaZYP1 *gene was used as the template for the synthesis of an α-^32^P dCTP labelled probe that was hybridised to a Chinese Spring nullisomic-tetrasomic membrane as per Lloyd *et al*. [30]. Autoradiography films were developed using an AGFA CP1000 Developer (AGFA, Nunawading, VIC, Australia).

### Q-PCR

Q-PCR was conducted in triplicate according to Crismani *et al*. [31]. Amplification of products was completed using gene specific Q-PCR primers (see Additional file 1: Table S1). The optimal acquisition temperature for *TaZYP1 *was 79°C. Statistical analysis to determine the mean ± SE of the three replicate experiments was conducted with GenStat software (version 11.0, Numerical Algorithms Group).

### Protein analysis

The *TaZYP1 *insert within the pCR8/GW/TOPO vector was cloned into a pDEST17 expression plasmid (Invitrogen) according to the manufacturer's LR clonase protocol. BL-21 A1 *E. coli *were transformed with the pDEST17-*TaZYP1 *ORF vector, and protein production was induced with 0.4% L-(+)-arabinose (w/v) (Sigma-Aldrich, St Louis, MO, USA). Protein isolation and purification was performed using nickel-nitrilotriacetic acid (Ni-NTA) beads (Qiagen, Clifton, VIC, Australia) according to the manufacturer's extraction protocols. Sodium dodecyl sulfate polyacrylamide gel electrophoresis (SDS-PAGE) was performed using NuPAGE Novex 4-12% Bis-Tris 7 cm mini-gels (Invitrogen) according to the manufacturer's protocol. Staining and destaining of gels were performed as previously reported [32].

The identity of the recombinant *Ta*ZYP1 protein was confirmed by ion trap liquid chromatography-electrospray ionisation tandem mass spectrometry (LC-MS/MS). Gel slices containing the recombinant *Ta*ZYP1 protein were washed with 100 mM ammonium bicarbonate, dried, rehydrated with 100 mM ammonium carbonate and subjected to in-gel tryptic digestion. LC-MS/MS of the digested peptides was then conducted as reported by March *et al*. [33].

### Polyclonal antibody production

The amino acid sequence of *Ta*ZYP1 was assessed for hydrophobic and antigenic regions using Kite-Doolittle and Hopp plots. The peptide sequence chosen for peptide synthesis was: CLRAYHKEELQRIRS. The peptide was synthesised by Mimetopes (Mimetopes, Clayton, Victoria, Australia) and conjugated to keyhole limpet using a hemocyanin maleimidocaproyl-N-hydroxysuccinimide (MCS) linker. The *Ta*ZYP1 peptide antigen was delivered to Institute of Medical and Veterinary Science for immunisation of two rats (*Rattus rattus*). The antigen was dissolved in 1× PBS (10 μg μL^-1^), added with an equivalent amount of Freund's complete adjuvant (Sigma-Aldrich) and used for primary immunisation of two rats via subcutaneous injection. Three subsequent immunisations were administered in three-week intervals, with Freund's incomplete adjuvant (Sigma-Aldrich) added to the dissolved antigen in 1× PBS. All immunisation doses contained 200 μg of *Ta*ZYP1 antigen. Immune sera was collected 10.5 weeks after the first injection.

### Competitive DNA binding assay

Recombinant full length *Ta*ZYP1 extracted under native conditions was quantified using the Bradford assay [34]. Competitive DNA binding assays were conducted as described by Pezza *et al*. [35] with modifications as per Khoo *et al*. [36]. The DNA binding abilities of *Ta*ZYP1 was tested with ΦX174 circular single-stranded DNA (ssDNA) (virion) (30 μM per nucleotide) (New England Biolabs, Beverly, MA, USA) and ΦX174 linear double-stranded DNA (dsDNA) (RFI form *Pst1*-digested) (15 μM per base pair) (New England Biolabs).

### Fluorescence immunolocalisation

Fluorescence immunolocalisation of *Ta*ASY1 and *Ta*ZYP1 was performed as per Franklin *et al*. [37] and Boden *et al*. [14] with the following changes: anthers were fixed with 2% paraformaldehyde and cells permeabilised for 3 h. For detecting the localisation pattern of *Ta*ZYP1, a rat anti-*Ta*ZYP1 antibody (1:100) and an AlexaFluor^® ^488 conjugated donkey anti-rat antibody (1:50; Molecular Probes, Invitrogen) was used. Optical sections (90-120 per nucleus) of meiocytes were collected using a Leica TCS SP5 Spectral Scanning Confocal Microscope (Leica Microsystems, http://www.leica-microsystems.com/) equipped with an oil immersion HCX Plan Apochromat 63 ×/1.4 lens, a 405 nm pulsed laser and an Argon laser using an excitation wavelength of 468 nm. Images were processed using Leica Application Suite Advanced Fluorescence (LAS-AF; version 1.8.2, build 1465, Leica Microsystems) software to generate maximum intensity projections of each nucleus.

## Competing interests

The authors declare that they have no competing interests.

## Authors' contributions

KHPK performed all experimental procedures and drafted the manuscript. KHPK, JAA and AJA participated in the design of the study. JAA and AJA drafted the manuscript. All authors read and approved the final manuscript.

## Supplementary Material

Additional file 1 **Table S1 Detailed list of primers used in this study**. A combination of standard and 3' RACE PCR was used to amplify *TaZYP1 *while open reading frame (ORF)-targeting primers were used to isolate the ORF sequence for protein production. Nested primers were used to confirm the sequence of the full-length *TaZYP1 *ORF while another set was used to isolate the 561 bp probe used for the Southern blot hybridisation. Q-PCR primers were used to determine the expression profile of *TaZYP1 *in meiotic tissues of the wild-type, *ph1b *and *Taasy1 *knock-down mutant plants. Abbreviations: F1, forward 1; R1, reverse 1; 3'R F1, 3'RACE forward 1; cF, confirmation forward; cR, confirmation reverse; QF1, quantitative forward 1; QR1, quantitative reverse 1; SF1, Southern forward 1; SR1, Southern reverse 1. T_m _(°C) = melting temperature. Click here for file
