# Supplementary material for: The isolation and characterisation of the wheat molecular ZIPper I homologue, TaZYP1
Source: BMC Res Notes. 2012 Feb 18;5:106. doi: 10.1186/1756-0500-5-106 (PMC3305362; doi:10.1186/1756-0500-5-106)
Supplement: Additional file 1 — Table S1 Detailed list of primers used in this study. A combination of standard and 3' RACE PCR was used to amplify TaZYP1 while open reading frame (ORF)-targeting primers were used to isolate the ORF sequence for protein production. Nested primers were used to confirm the sequence of the full-length TaZYP1 ORF while another set was used to isolate the 561 bp probe used for the Southern blot hybridisation. Q-PCR primers were used to determine the expression profile of TaZYP1 in meiotic tissues of the wild-type, ph1b and Taasy1 knock-down mutant plants. Abbreviations: F1, forward 1; R1, reverse 1; 3'R F1, 3'RACE forward 1; cF, confirmation forward; cR, confirmation reverse; QF1, quantitative forward 1; QR1, quantitative reverse 1; SF1, Southern forward 1; SR1, Southern reverse 1. Tm (°C) = melting temperature. [file 1756-0500-5-106-S1.DOC]

**Supplementary Information File 1**

Khoo KHP, Able AJ & Able JA (2011).

The isolation and characterisation of the wheat Molecular ZIPper I homologue, *Ta*ZYP1

**Table S1 - Detailed list of primers used in this study.**

A combination of standard and 3′ RACE PCR was used to amplify *TaZYP1* while open reading frame (ORF)-targeting primers were used to isolate the ORF sequence for protein production. Nested primers were used to confirm the sequence of the full-length *TaZYP1* ORF while another set was used to isolate the 561 bp probe used for the Southern blot hybridisation. Q-PCR primers were used to determine the expression profile of *TaZYP1* in meiotic tissues of the wild-type, *ph1b* and *Taasy1* knock-down mutant plants. Abbreviations: F1, forward 1; R1, reverse 1; 3′R F1, 3′RACE forward 1; cF, confirmation forward; cR, confirmation reverse; QF1, quantitative forward 1; QR1, quantitative reverse 1; SF1, Southern forward 1; SR1, Southern reverse 1. T_m_ (°C) = melting temperature.

| **Primer name** | **Primer sequence (5´ → 3´)** | **T_m_ (°C)** |
| --- | --- | --- |
| ***TaZYP1* gene isolation primers** | | |
| *TaZYP1*_F1 | ATGCAGAAGCTGGGTTTATCGGG | 60 |
| *TaZYP1*_R1 | AATGCTCCTTGCTTCTCCTCCTTTGACT |
| ***TaZYP1* 3′ RACE primers** | | |
| *TaZYP1_*3′R F1 | TCAGAAGTCAAAGGAGGAGAAGCAAAGAGCATT | 62 |
| GeneRacer™ 3′ Primer | GCTGTCAACGATACGCTACGTAACG | 76 |
| GeneRacer™ 3′ Nested Primer | CGCTACGTAACGGCATGACAGTG | 72 |
| ***TaZYP1* gene expression primers (to amplify ORF for protein production)** | | |
| *TaZYP1*_ORF_F1 | ATGCAGAAGCTGGGTTTATCGGG | 58 |
| *TaZYP1*_ORF_R1 | CTAGGCAAATGCATAAGGGTCATCAGC |
| ***TaZYP1* nested primers (to obtain full-length *TaZYP1* sequence)** | | |
| *TaZYP1*_cF1 | ACAGTTGGAGGGTTCAGTTGAAGA | 55 |
| *TaZYP1*_cR1 | GCCTCGGTAAGTTGACATTCTG |
| *TaZYP1*_cF2 | ATCCCGCTTATTGTGTGCTGACT | 55 |
| *TaZYP1*_cR2 | CCTGAAGCATGAGATCGTACTGTT |
| **Plasmid vector sequencing primers** | | |
| GW1 (pCR®8/GW/TOPO®) | GTTGCAACAAATTGATGAGCAATGC | 50 |
| GW2 (pCR®8/GW/TOPO®) | GTTGCAACAAATTGATGAGCAATTA |
| T7 (pDEST17) | TAATACGACTCACTATAGGG |
| **Quantitative real-time PCR (Q-PCR) primers** | | |
| *TaZYP1*_QF1 | GCTTCAGTTGCCAGGTCCAG | 57 |
| *TaZYP1*_QR1 | CAATGACTTCTGAGTATTCGGTTCC |
| **Southern blot probe primers** | | |
| *TaZYP1*_SF1 | ACAAAAGTTACAGATCCAAGCATCA | 58 |
| *TaZYP1*_SR1 | AATGCTCTTTGCTTCTCCTCCTTTGACT |
